# Data for tracking SDGs: challenges in capturing neonatal data from hospitals in Kenya

**DOI:** 10.1136/bmjgh-2019-002108

**Published:** 2020-03-31

**Authors:** Christiane Hagel, Chris Paton, George Mbevi, Mike English, on behalf of the Clinical Information Network (CIN) information systems interest group

**Affiliations:** 1 Nuffield Department of Medicine, Centre for Tropical Medicine and Global Health, University of Oxford, Oxford, United Kingdom; 2 KEMRI-Wellcome Trust Research Programme, Health Services Unit, Nairobi, Kenya

**Keywords:** health policy, health systems, paediatrics

## Abstract

**Background:**

Target 3.2 of the United Nations Sustainable Development Goals (SDGs) is to reduce neonatal mortality. In low-income and middle-income countries (LMICs), the District Health Information Software, V.2 (DHIS2) is widely used to help improve indicator data reporting. There are few reports on its use for collecting neonatal hospital data that are of increasing importance as births within facilities increase. To address this gap, we investigated implementation experiences of DHIS2 in LMICs and mapped the information flow relevant for neonatal data reporting in Kenyan hospitals.

**Methods:**

A narrative review of published literature and policy documents from LMICs was conducted. Information gathered was used to identify the challenges around DHIS2 and to map information flows from healthcare facilities to the national level. Two use cases explore how newborn data collection and reporting happens in hospitals. The results were validated, adjusted and system challenges identified.

**Results:**

Literature and policy documents report that DHIS2 is a useful tool with strong technical capabilities, but significant challenges can emerge with the implementation. Visualisations of information flows highlight how a complex, people-based and paper-based subsystem for inpatient information capture precedes digitisation. Use cases point to major challenges in these subsystems in accurately identifying newborn deaths and appropriate data for the calculation of mortality even in hospitals.

**Conclusions:**

DHIS2 is a tool with potential to improve availability of health information that is key to health systems, but it critically depends on people-based and paper-based subsystems. In hospitals, the subsystems are subject to multiple micro level challenges. Work is needed to design and implement better standardised information processes, recording and reporting tools, and to strengthen the information system workforce. If the challenges are addressed and data quality improved, DHIS2 can support countries to track progress towards the SDG target of improving neonatal mortality.

**Key questions**

**What is already known?**

- Data are needed to track progress towards Target 3.2 of the Sustainable Development Goals (SDGs) to end preventable deaths of newborns.
- District Health Information Software, V.2 (DHIS2) is a widely used data platform in low-income and middle income countries (LMICs) which forms part of the health information system in LMICs.
- Almost a decade after the national roll-out of DHIS2 in Kenya, it seems timely to examine how the information system is working to support neonatal data collection and reporting.

**What are the new findings?**

- Use cases from Kenyan hospitals show that the neonatal information flow to DHIS2 is suboptimal with a corresponding lack of confidence in the quality of data.
- Digitisation is based on a people-based and paper-based subsystem in Kenya before data are uploaded to DHIS2 by Health Records Information Officers meaning the staff on the wards and the systems put in place to co-ordinate data flow are critical components of the health information system.
- While DHIS2 is technically able to support collection of hospital data as part of tracking progress towards SDG 3.2, failures in the organisation and personnel subsystems mean this is currently unlikely to be achieved.

**What do the new findings imply?**

- The national roll-out of DHIS2 has been a first technical step towards rationalising and harmonising different subsystems and databases.
- The practice of morbidity and mortality reporting for both healthy and sick newborn babies remains a serious challenge.
- Considerable work is needed to address weaknesses in the design of the organisation and personnel subsystems, including a significant investment in the information workforce, to improve neonatal outcome reporting from hospitals.

## Background

When the United Nations General Assembly launched 13 targets and 28 indicators in 2015 for the 2030 Sustainable Development Goals (SDGs), the neonatal mortality rate was defined as a specific indicator (SDG 3.2.2).^1^ Target 3.2 recognises that almost 50% of all deaths of children under 5 occur in the neonatal period (the period between birth up to 1 month of life) in low-income and middle-income countries (LMICs).^2–5^ In order to track progress towards national and global goals and equity gaps, reliable neonatal data are needed for decision-makers at local, national, regional and global levels that are high quality, timely, accessible and easy to use.^6–8^

District Health Information Software, V.2 (DHIS2) is a widely used data platform designed to improve health information systems in LMICs in general, and indicator data reporting in particular through routine systems.^9 10^ Kenya was one of the first countries to implement DHIS2 on a national scale in 2010.^9^ Almost a decade after the national roll-out of DHIS2, it seems timely to examine how the information system is working to support the neonatal data collection needed to track progress towards SDG 3.2.2. We focused in particular on neonatal data collection in hospitals because this is where an increasing number of births take place and almost 50% of neonatal mortality occurs within 24 hours of birth in LMICs.^11^ We use the term newborn mortality hereafter to mean in-hospital mortality of babies born in the facility. These events are typically focused in the first days of life.

Our aims were

1. To investigate implementation experiences of DHIS2 in LMICs with a specific focus on identifying general challenges and those particular to hospitals.
2. To map the data collection and reporting processes for capturing in-hospital newborn mortality in Kenyan hospitals and identify potential challenges in the production of quality data.

## Methods

### Setting: specifics of the Kenyan context using DHIS2

DHIS2 was piloted in Kenya in 2010 and subsequently rolled out nationwide in 2011 as the government’s national health data reporting system.^10 12^ DHIS2 is a software system for data collection, validation, analysis and presentation of statistical data tailored to health information management that is a key tool in many countries’ wider health information management system or health information system. DHIS2 can cover aggregated data (eg, routine health facility data, staffing, equipment, etc) and capture events (eg, disease outbreaks, patient satisfaction surveys, etc). It is flexible, adaptable and extendable through web application program interfaces (Web APIs), which are used for building linked software applications. The software works offline in times of electricity shortages or a lack of internet connectivity and provides inbuilt data validation.^9 10^ While DHIS2 is open source and free of license fees, other costs include training, set-up, implementation and maintenance.

**Figure 1:**
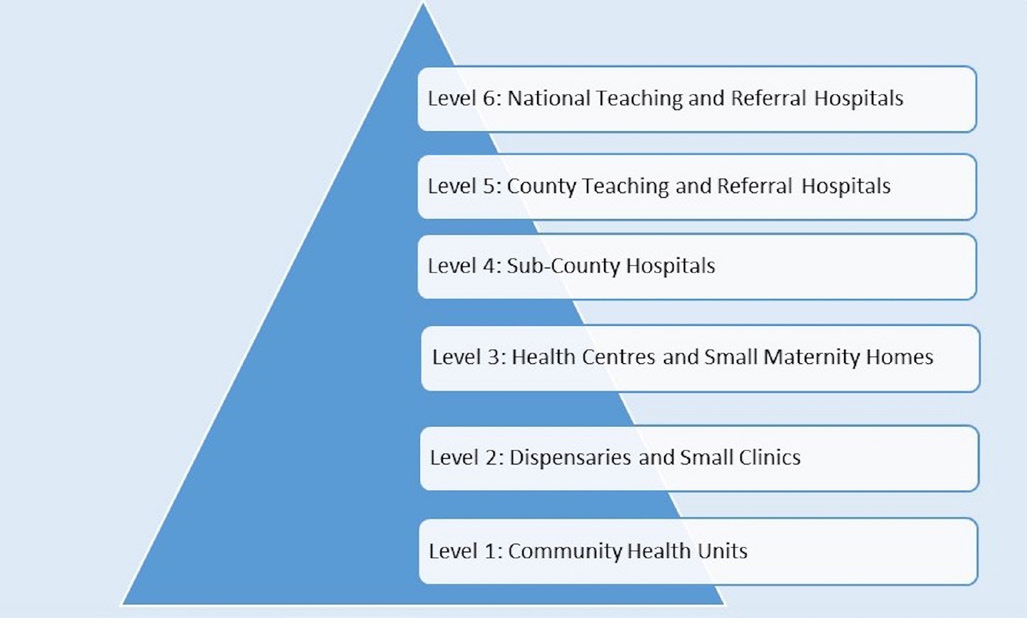
Healthcare levels in Kenya.

During the implementation of DHIS2, Kenya devolved all responsibility for health service delivery to each of the 47 Kenyan counties that make up the country. The national government remains responsible for policy-making, quality assurance and standards, and monitoring and evaluation while the county governments are responsible for the management of county health facilities (including their health information system functions), and disease surveillance and response.^13–15^ There are six levels of service delivery in Kenya (see figure 1) where health data collection occurs.^14 15^ Our work was primarily focused on levels 4, 5 and 6 hospitals of this system that provide delivery care to large numbers of mothers and where research studies suggest newborn mortality is high.^16 17^ Health Records and Information Officers (HRIOs) in Kenya have data entry rights and are responsible for entering data to the DHIS2 system at facility or sub-county level.

### Narrative review of literature on DHIS2 implementation experiences and policy documents to characterise the architecture of the information system in Kenya

For aim 1, we conducted a narrative review based on structured search strategies of published research and policy documents with the purpose of identifying the challenges and successes of DHIS2 implementation in general and any specific reports on DHIS2 implementation in Kenya.^18 19^

Policy documents were identified by searching through official governmental websites and through personal communications with people working in this area. Information was used to describe the intended data collection workflow from facilities up to the global level. This process involves both paper-based and electronic information flows visualised in the form of a flow diagram.^20^ These were validated and adjusted based on discussions with key informants, including HRIOs from six different levels 4–6 hospitals and one sub-county HRIO, and officials from the “Division of Monitoring and Evaluation (M&E), Health Research Development & Health Informatics” of the Kenyan Ministry of Health (MoH) (see online supplementary file 1 for search strategy, PRISMA diagram and flow diagram mapping steps).

10.1136/bmjgh-2019-002108.supp1 Supplementary data

### Developing ‘use cases’ to map data capture specific to newborn mortality in hospitals

For aim 2, we developed two descriptive, explanatory ‘use cases’, a system analysis method to “identify, clarify, and organise requirements in a system”,^21^ in order to explore the micro level approaches to capturing newborn morbidity and mortality data at hospital level in LMICs using Kenya as an example. These were based on detailed examination of events on the ground and discussions with key informants. Hospitals’ senior nurses and HRIOs who are most directly involved in the processes helped us to validate and adjust our generated use cases and mappings.

The following two information use cases were investigated: (1) healthy neonates, born in the hospital; and (2) sick neonates, born in the hospital who required immediate inpatient care. Neonates born outside of the hospital were not included as admission of these newborns is less common and policies guiding admission vary widely between hospitals.^22^ For the use cases, we documented and visualised the data collection and reporting process to DHIS2 spanning four newborn events: (1) *delivery*, which happens usually within the maternity (labour) ward for normal delivery or the operating theatre for caesarean sections; (2) *postnatal/neonatal care*, which happens at the postnatal ward if the baby is healthy or at the Newborn Unit (NBU) if the baby is sick; (3) *immunisation* which begins within days of birth in many LMICs including Kenya and which happens typically at exit from the NBU for sick babies or at the outpatient/maternal and child health clinic for those who were healthy and discharged; and (4) *discharge, referral or death*. Use cases were validated and adjusted based on discussions with the hospitals’ senior nurses and HRIOs.

**Figure 2:**
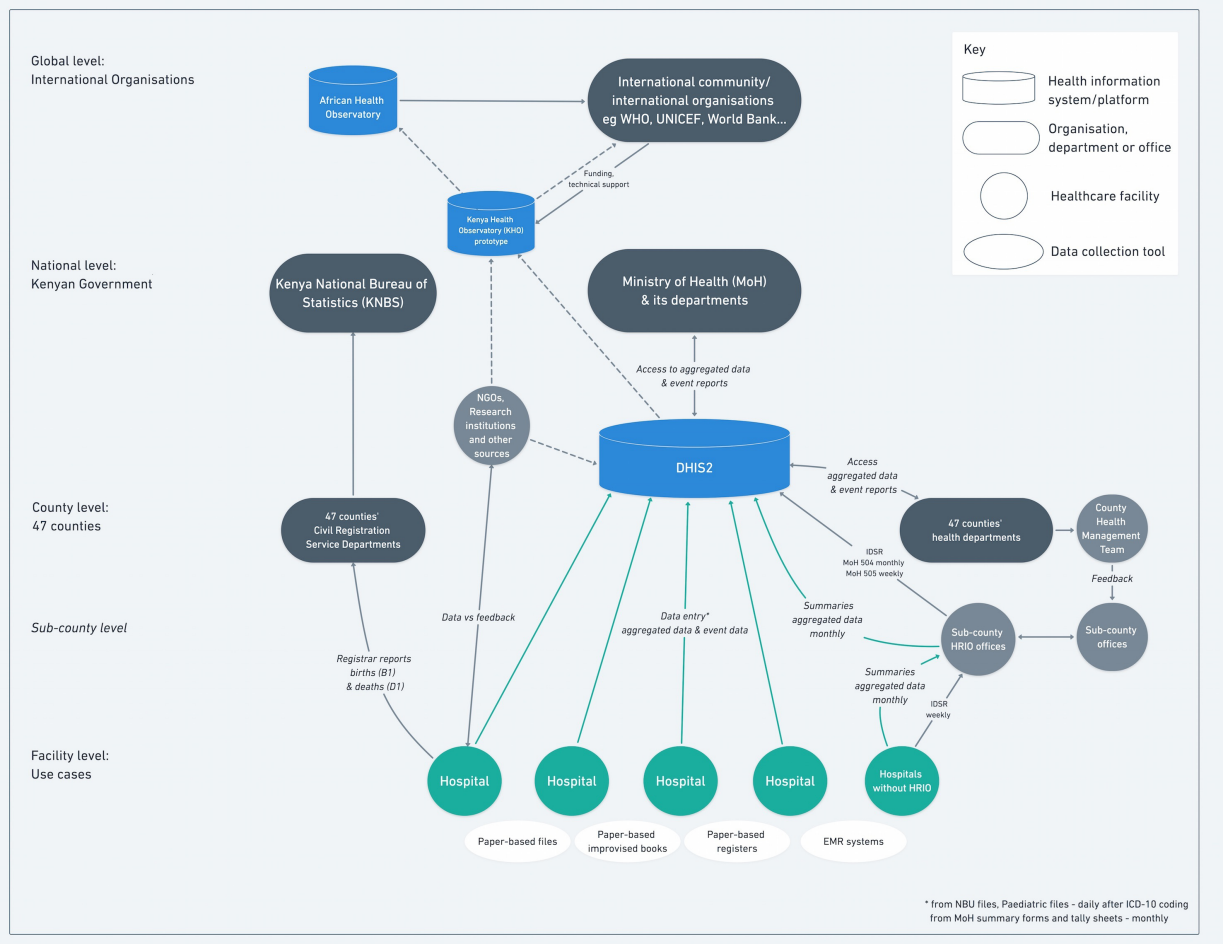
Health information flow to DHIS2 in Kenya.

**Figure 3:**
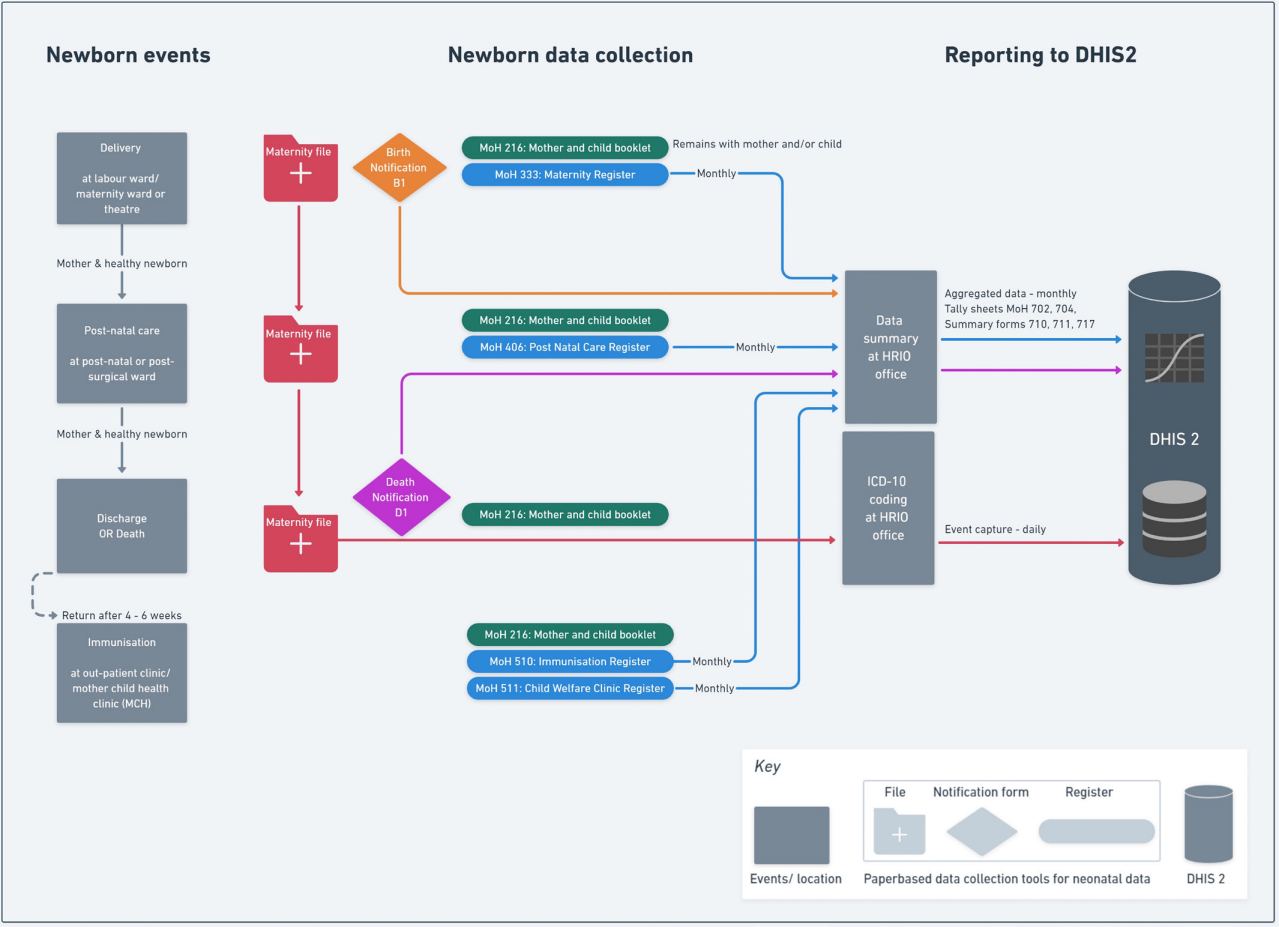
Use case 1 for healthy inpatient neonates.

**Figure 4:**
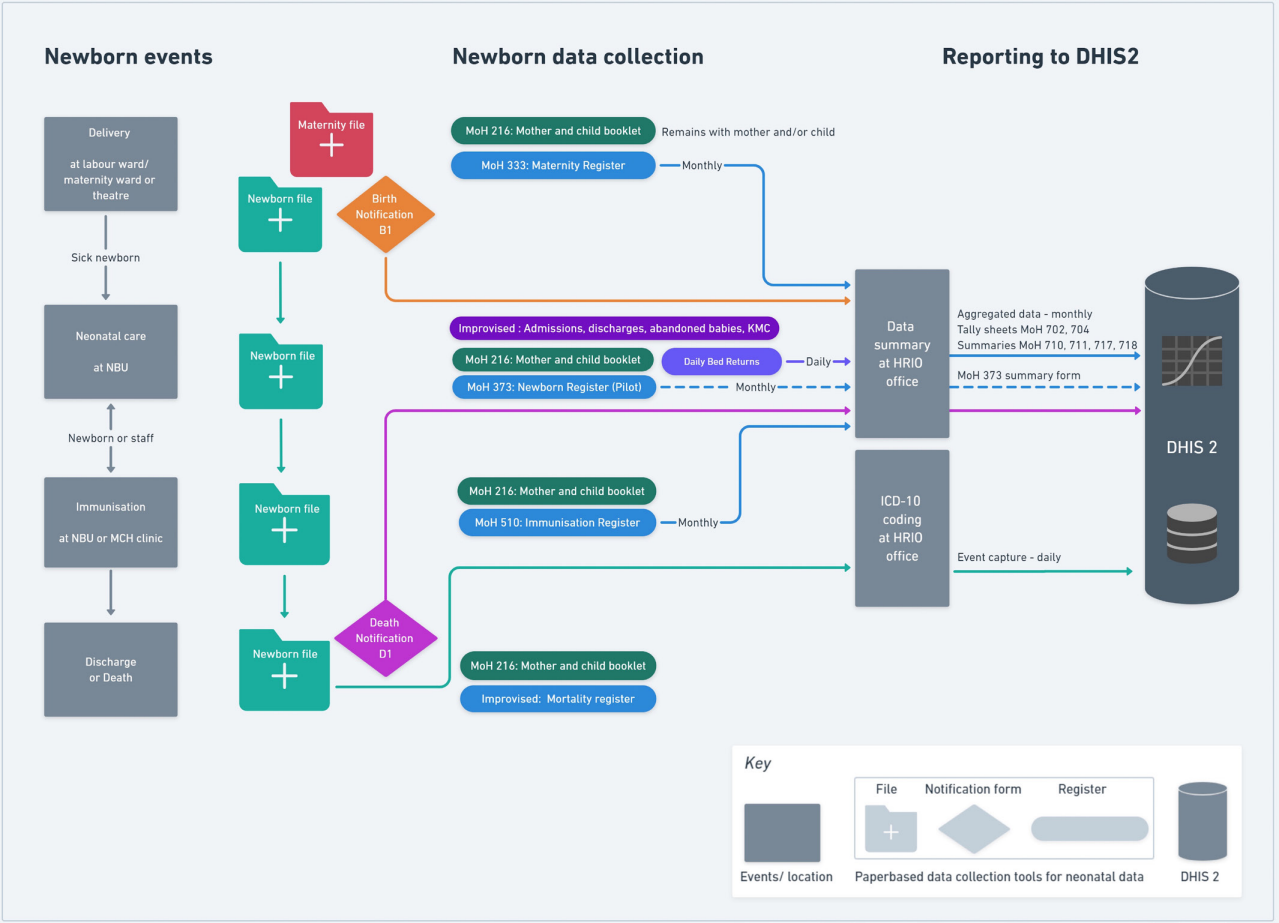
Use case 2 for sick inpatient neonates admitted to a Newborn Unit (NBU).

For visualisations (figures 2–4), the Whimsical.co visualisation tool was used.^13^ This research was done without patient or public involvement as the focus was mainly on the health information system and its infrastructure. The discussions with key informants were conducted in order to validate our findings from the literature (and observations) rather than as a structured interview. We started discussions with one question “Is what you see in our visualisations correct and, if not, how would you correct it based on your experience”. In addition, we asked for permission to look at the different forms in use. For both, we asked the hospital management and key informants for permission and assent.

## Results

For aim 1, we separate our findings into two sections. First, we highlight findings from the published research and grey literature on DHIS2 that point to the wider implementation challenges in generating high-quality data to track health indicators (section 1). Second, we present the planned Kenyan architecture of DHIS2 (section 2). With this background, we then focus on the findings for aim 2 with our detailed newborn use cases (section 3).

### Challenges implementing DHIS2

We examined 17 research articles (see online supplementary file 2 (a)) that had a specific focus on DHIS2 in Kenya and identified 67 that reported experiences from other LMICs. From these, we identified one recent literature review by Dehnavieh *et al* that reports the strengths and operational challenges of DHIS2,^23^ and we build on this using results from the additional studies we found.

10.1136/bmjgh-2019-002108.supp2 Supplementary data

Globally, the Dehnavieh *et al* review and the wider research and grey literature consistently report that while DHIS2 has strong technical capabilities to support health data collection in LMIC settings, there are also significant operational and organisational challenges in its implementation.^9 23–28^ Challenges at a macro level include workforce capacity, adequate financing and the political, social and infrastructural context.^23 24^ Access to computers and appropriate ICT infrastructure remain operational challenges for DHIS2 in LMICs, including Kenya.^23 29^ Also, there have been meso level and micro level challenges reported. These include, for example, the collection, collation, compilation and reporting of health data by personnel at the facility level. These result in poor quality data which undermine their value for decision-making. While some specific evaluations conclude that data quality can be acceptable,^30^ many do not span the recommended six dimensions of accuracy, validity, timeliness, uniqueness, consistency and completeness.^31 32^

In Kenya as in many countries, DHIS2, as it is deployed, is used for capture of pre-aggregated data. The primary data capture and aggregation depends on a number of physical registers and paper-based files and thus on the documentation efforts of ward-based healthcare workers (HCWs) and HRIOs (see use cases 1 and 2). Digitisation of health data then begins with the summary data upload to DHIS2 by HRIOs.^33 34^ The use of paper-based health information systems as a precursor to digital data capture is the norm in sub-Saharan African hospitals.^26 35 36^ Studies on DHIS2 specific to Kenya show it is ‘technically sound’ and can be successfully deployed and has improved data accessibility and completeness of reporting.^37–39^ Challenges reported in Kenya, however, include data quality (as reported globally), lack of standardisation, and the overload of data elements and indicators^40^ when healthcare or information system personnel already have high workloads.^41^ Indeed, DHIS2 as implemented in Kenya is highly dependent on the HRIOs who have rights to enter data from paper-based systems. According to Kuyo *et al*, they are “the main champions promoting the use of DHIS2 information” but absence of trained staff, poor skills among users and inadequate management support are major challenges in using DHIS2 effectively.^24^

### Kenyan information system architecture

We reviewed 23 documents (policies, strategies, assessment reports and guidelines on DHIS2 or concerned with information technology use in health more widely) from between 2010 and 2018 (see online supplementary file 2 (b)). These clearly show the investment the Kenyan government has made in developing the strategic and regulatory environment for Kenya’s health information system, the transition to the use of digital tools and a commitment to better health facility reporting. This includes Kenya’s ambition to create a Kenyan Health Observatory, an online repository for health data, research data and knowledge products, which shall complement the African Health Observatory providing information for the international community.^42^ We provide a representation of the health information system using DHIS2 in Kenya as it is desired in figure 2.

The policies state that all hospital data from purpose-designed paper registers (see table 1) should be entered into the DHIS2 system in the form of summary tables and that data from system levels (health facilities, county up to the national level) should be collated, analysed, disseminated, and used for feedback and decision-making. Hospitals’ HRIOs are expected to review their previous month’s reports by the 16th day of the following month for quality checks. Any issues should be discussed and any errors identified should be corrected.^43^ Policy documents acknowledge that data quality (in all their dimensions) is a serious challenge. In 2014, the MoH reported that only a third of hospitals were submitting service delivery reports to sub-counties, counties and to the national level,^44^ that data sharing inside the facilities was lacking, that interoperability between facilities and systems is a major challenge, and that there is limited workforce capacity.^45–47^

**Table 1:** Paper-based forms in use for collecting newborn related data; partly usable as legend for figures 2–4

Type	Description
Notification forms	B1—Birth notification
	D1—Death notification
Health record booklet	MoH 216—Mother and Child Booklet—*remains with mother and/or child*
Index card	MoH 268—Inpatient Diagnostic Disease Index—*rarely in use*
Registers*	MoH 301—Inpatient Register—*general inpatient register, improvised for use in NBU*
	MoH 333—Maternity Register
	MoH 373—Inpatient Neonatal Register—*pilot*
	MoH 406—Post Natal Care Register
	MoH 510—Immunisation Permanent Register
	MoH 511—Child Welfare Clinic Register
Tally sheets	MoH 702—Immunisation Tally Sheet
	MoH 704—Child Health and Nutrition Information System (CHANIS) Tally Sheet for Child Health Welfare Clinics
Summary forms	MoH 373—Inpatient Neonatal Summary Form—*pilot*
	MoH 504—Integrated Disease Surveillance and Response (IDSR)—*monthly report*
	MoH 505—Integrated Disease Surveillance and Response (IDSR)*—weekly report*
	MoH 710—Immunisation Services Uptake Summary
	MoH 711 A and B—Integrated Reproductive and Child Health Summary
	MoH 717—Monthly Workload Report for Health Facilities
	MoH 718—Inpatient Morbidity and Mortality Summary
Other hospital forms	Facility/ward internal transfer form
	Daily bed return (DBR) form
Improvised forms or records	Newborn Unit (NBU) Admissions Record
	NBU Discharge/Exit Form
	Mortality and Cause of Death Register
	Kangaroo Mother Care (KMC) Register—*sometimes provided by NGOs*
	Abandoned Babies Register

*All registers contain individual data, but none of this individual data is entered into DHIS2 at present except as summary (aggregate), see figures 3 and 4.

### Facility-level newborn use cases

#### Use case 1: information about healthy neonates born in hospitals

For a healthy neonate that is born in the facility (use case 1), the information flow for the four newborn-related events/information collection points outlined in the methods section are summarised in figure 3. All births at the labour ward, maternity ward or theatre are supposed to be documented with the birth notification forms (B1) that are collected by the HRIOs, with information then forwarded to the Registrar of births and deaths of the Kenya National Bureau of Statistics as part of the Civil Registration and Vital Statistics (CRVS) system. This information is not entered into DHIS2. Information for the healthy newborn may be available in the mother’s hospital admission/maternity file. When a mother is discharged, these files are sent to the HRIO’s office where coding for the mother’s admission event (eg, diagnosis) is conducted, a process that might, in theory, enable capture of documented newborn events (live birth, stillbirth or death before discharge). Use case 1 illustrates, however, that the paper-based maternity ward registers provided by the MoH are the primary source for the summary report forms on numbers of healthy babies born in the facility (see table 1). From these registers, data summaries (extracted from ward summary forms and tally sheets) are submitted as aggregated data to DHIS2 on a monthly basis. Hospitals do not open a specific (inpatient) file for healthy or stillborn babies and as a result neither healthy newborns nor stillbirths are issued with a hospital inpatient number as a form of unique patient identifier and no standard discharge document for these individual babies is retained by the hospital. For babies who return for later immunisation visits or with a severe illness to the outpatient ward, there is no ability to clearly link these events to the birth record.

If a baby is born apparently healthy, the mother and baby will typically be transferred to a postnatal ward. While a later maternal complication (eg, postpartum sepsis) would be captured as a morbid episode in the mother’s hospital file, postnatal events affecting the baby will not typically be captured in any record unless the baby is admitted to the inpatient NBU (see below, use case 2). If the baby dies suddenly without admission to the NBU, then capturing this event may, in theory, occur through one of two mechanisms. Either there is an effort to update the original maternity ward register entry or a death notification form (D1) is completed for the baby that certifies the cause of death including antecedent causes and underlying cause.^48^ Information from local respondents suggests that postnatal ward staff would rarely update the maternity register that belongs to a functionally distinct team. Capturing this neonatal death through the D1 form requires the HRIO to include the death—which will likely not be clearly linked to a specific register entry—while they are using the maternity register to create the monthly summary DHIS2 table. Again, local reports suggest this ad hoc adjustment is variably performed.

#### Use case 2: information about sick neonates

Use case 2 illustrates the information flow for sick neonates (see figure 4). Data for sick babies are collected at the same four points as for healthy babies, but immunisation usually happens during care on the NBU just before discharge. The main addition and difference is that a separate inpatient file is opened for sick neonates to support clinical care on the NBU. This newborn file is physically taken to the coding office after discharge or death and data on newborn mortality and morbidity can, in theory, be submitted to DHIS2 as a summary of specific NBU ward activity. In the case of a death occurring on the NBU a D1 form (as discussed above) is also completed. However, as above, these morbidity and mortality NBU events cannot be linked to any of the mother’s data. Furthermore, in the Kenyan hospitals studied, there was in fact no nationally recommended paper NBU summary data form and no corresponding operational DHIS2 summary data capture table (a challenge now being addressed). In fact, in NBUs, there were several improvised (sometimes also called ‘informal’) paper-based registers used in parallel. These included admission books, discharge books, registers for Kangaroo Mother Care (KMC) or abandoned babies often used because MoH registers were not supplied in time, were abolished or did not collect information needed by the HCWs. Data contained in such informal registers are not reviewed by the HRIO or used in any DHIS2 reporting procedure.

#### Practical information problems in both use cases

By building both use cases, we were able to identify multiple practical information problems at the micro level, in particular for those newborns who do not have their own inpatient file:

1. *Record classification:* An initially healthy newborn recorded as alive in a maternity register, who dies and who never has its own inpatient file, raises two issues. First, the death may never be recorded as it relies on HCWs or HRIOs updating the maternity register data. The postnatal register (MoH 406) does not specifically record neonatal deaths. Second, if an HRIO identifies the death of a baby postnatally, the ability to infer the cause of death relies on any documentation of the baby’s illness in the mother’s medical record.
2. *Documentation consistency/ stillbirth misclassification:* There exist significant inconsistencies from facility to facility as to which documents are used to collect and report newborn data to DHIS2, especially for stillbirths. For a healthy or stillborn baby, it is most likely that data are recorded in the maternity register (MoH 333) as it asks specifically if the baby is born dead and collects basic data for the healthy newborn. However, if the baby dies rapidly after delivery on the maternity unit (eg, because resuscitation fails), there is no consistency in whether it is recorded as a stillbirth or a neonatal death and therefore neonatal deaths can sometimes be misclassified as stillbirths. From one key informant, we learnt that this might be a result of the current complex paper-based system in use creating confusion about which form should be used. And since it appears to be easier to record this case as a stillbirth rather than a neonatal death (as it avoids filling out D1 and proceeding to a neonatal audit), it may result in over-reporting of stillbirths and under-reporting of neonatal deaths.
3. *Record linkage:* The challenges previously mentioned result in inconsistencies and misreporting of neonatal mortality and morbidity events among in-born babies. Larger facilities may also admit babies born elsewhere (either at home or at smaller facilities) to their NBU, some of whom may die. These mortality and morbidity events can incorrectly inflate the numerator when estimating the in-facility newborn mortality rate as this is typically calculated using a denominator of in-facility live births.

## Discussion

We discuss first the implications of our study of use cases (aim 2) before returning to discuss issues affecting the broader information landscape (aim 1). We do this to emphasise that we must solve problems with neonatal data capture at the micro level if we are to gain the benefits of investments to information systems more widely to support tracking of neonatal mortality rates as part of monitoring progress towards the SDGs.

### Neonatal information flow challenges at the micro level

#### Newborn mortality reporting from hospitals

Reporting of births and deaths, in particular of stillbirths, remains a serious challenge. With the tools currently in use, it may be easier to record a very early neonatal death as a stillbirth. Kenya is introducing a new neonatal inpatient register (MoH 373) for NBUs designed to report, among other indicators, newborn mortality to DHIS2. It will, however, only capture data for those babies sick enough to be admitted to the NBU. There is no standardised system for aggregating all stillbirths and neonatal deaths occurring on the maternity or postnatal wards and the NBU, which is both comprehensive and avoids double counting. Instead, misclassification of stillbirths occurs and existing systems do not enable rates to be reported of newborn deaths in the facility for babies born in the same facility. More broadly, there are two parallel paths for reporting neonatal deaths, one for the health information system (DHIS2) and one for vital registration (CRVS run by the National Bureau of Statistics). Events in these systems are not currently linked.

#### Newborn morbidity reporting

Beyond neonatal mortality, it is critical to have reliable neonatal morbidity information. For babies admitted to NBU, the new newborn register (MoH 373) is designed to capture morbidity events and enable reporting to DHIS2. Postnatal events affecting the initially healthy baby will still not be systematically collected unless a baby is admitted to a NBU (use case 1). Thus, information on mild morbidity (eg, jaundice or congenital abnormalities such as talipes, etc) will not be captured. One possible solution to capturing both better mortality and morbidity data is that all babies (healthy or sick) that are born within the facility, admitted from outside the facility, or stillborn could be issued with a unique hospital number and record, ideally linked to information on the mother and the place of birth. This then could become the primary source document for facility-based newborn mortality and morbidity reporting. This would, however, further increase the work of HRIOs.

#### The role of staff

The use cases (figures 3 and 4) revealed that digitisation begins usually with data uploaded to DHIS2 through the HRIO who needs to collect and aggregate data from different paper-based sources. In such people-based and paper-based subsystems, appropriate training, time and support is needed for healthcare staff and HRIOs to be able to record data carefully and then transfer it into DHIS2. Electronic Health Record (EHR) systems can in theory support better data collection, but they remain costly and challenging to implement and sustain.^49 50^ Initially, therefore, supplies of basic registers and inpatient files that are often lacking need to be improved while development of additional standardised, co-designed paper-based records might help support routine work and replace the informal, improvised registers (such as KMC registers, Mortality and Cause of Death or NBU Admissions and Discharge books) staff have already introduced. Better understanding of how health workers use information and how records flow, as illustrated in figures 2 and 3, may then support the design of EHR that suit the context.

### Broader health information system challenges

The national roll-out of DHIS2 was a significant step towards rationalising and harmonising different subsystems and databases in Kenya.^51 52^ As health information systems are acknowledged as one of the core blocks of health systems,^53^ they are expected to collect quality data and to convert data into reliable information for decision-making to improve health services,^39 49^ and support routine clinical work, for example, through feedback to staff about outcomes.^54 55^ To do so, the Kenyan health information system needs further improvements, as acknowledged in a recent MoH report, suggesting data capture and utilisation needs to be strengthened at all levels.^40^ While DHIS2 has helped to improve quality of data in some dimensions (eg, timeliness and completeness) and where data capture is simple, for example, clinic visits, vaccines and immunisation events,^29 40 56^ there remain significant challenges in capturing more complex facility-based data as we illustrate in this article.

### Limitations

We used a narrative review rather than a formal systematic review to understand the broader health information system context and DHIS2 implementation but included a large number of source documents and do not feel that additional literature would have changed our findings significantly. Characterising the Kenyan health information system relied on grey literature and governmental websites. It can be problematic to identify all the relevant documents and these might not be updated regularly. We used key contacts to help us to identify the most relevant and up-to-date documents. We specifically focused on information capture and flows into the DHIS2 system. This excludes other important aspects of information systems such as the processes of data management, analysis and use in decision-making. Our rationale being that these processes rely on accurate data capture. In order to build generic use cases (figures 3 and 4), we excluded from our study other important groups, such as newborn admissions to hospital after birth at home or in smaller facilities. We looked at information flows only at the hospital level, missing out the community level and events outside hospitals. Thus, our research does not explore how data on the large number of home deliveries might be captured or integrated with facility-based data in the current health information system. Neonatal data collection for HIV treatment or visits to clinics with illness episodes within the first month of life were also excluded.

## Conclusion

DHIS2 is a tool with the potential to improve the availability and use of health data. The results of our literature review and information flow mapping demonstrate that capturing in-hospital newborn data wit DHIS2 depends on a complex people-based and paper-based subsystem in Kenya. Challenges identified mean that even from hospitals, neonatal data collection and reporting processes to DHIS2 are suboptimal with a corresponding lack of confidence in the quality of data that DHIS2 provides. Subsystem tools and processes at the micro level should be improved, probably using co-design with hospital staff so that data capture takes account of their different perspectives, routines, skills and information needs. The information workforce also needs to be expanded. If the challenges identified in the information subsystems are addressed and data quality improved, DHIS2 has the potential to become a valuable tool for supporting countries to track their progress towards the SDG 3.2 target of improving neonatal mortality.
