# Supplementary material for: Data for tracking SDGs: challenges in capturing neonatal data from hospitals in Kenya
Source: BMJ Glob Health. 2020 Mar 31;5(3):e002108. doi: 10.1136/bmjgh-2019-002108 (PMC7170465; doi:10.1136/bmjgh-2019-002108)
Supplement: Supplementary data [file bmjgh-2019-002108supp001.pdf]

Supplementary file 1: Search strategies (a), PRIMSMA diagram (b) and information flow diagram (c)

(a) Literature search strategy

The search for pivotal literature and reports on DHIS2 compromised two steps (last update: April 2019). Relevant research articles were searched in the Clarivate Web of Science database (all databases) and PubMed (United States National Library of Medicine) using different keyword strategies. Due to the high volume of findings, results from search # 1-3 and results from Google Scholar were only cross-checked for additional results, meaning only the first 50-100 results sorted by relevance were reviewed. Results for search # 4-7 were considered for review and duplicates deleted (manually and automatically using Mendeley software as reference manager). All remaining studies were reviewed in four steps: title, author keywords, abstract and full text. Publications were selected that were considered relevant for this study in that they would provide information on the key lessons using DHIS2 with a priority on studies in Kenya. In addition, grey literature and reports of relevant stakeholder organisations were cross-checked and identified by searching through websites from international organisations and their partnerships.

#	Search	Web of Science – All Databases	PubMed	Google Scholar for cross-check
**1**	Year range: none<br>Keywords (Topic): (DHIS 2) OR (DHIS2) OR (District Health Information Software 2) OR (District Health Information System 2)	2,595	1,389	1,670
**2**	Year range: 1996 – 2019<br>Keywords (Topic): (DHIS 2) OR (DHIS2) OR (District Health Information Software 2) OR (District Health Information System 2)	2,302	1,296	1,630
**3**	Year range: 1996 – 2019<br>Keywords (Topic): DHIS2 OR (District Health Information Software 2)	295	184	1,350
**4**	Year range: 1996 – 2019<br>Keywords (Topic): DHIS2	58	23	1,320
**5**	Year range: 1996 – 2019<br>Keywords (Topic): (DHIS2 OR (District Health Information Software 2)) AND implementation	48	40	894
**6**	Year range: 1996 – 2019<br>Keywords (Topic): (DHIS2 OR (District Health Information Software 2)) AND (data quality)	50	30	880
**7**	Year range: 1996 – 2019<br>Keywords (Topic): (DHIS2 OR (District Health Information Software 2)) AND ((DHIS2 data use) OR (DHIS2 data usage) OR (DHIS2 data utilization))	29	23	560
	**Publications considered for review #4-7**	**185**	**116**	
	**Publications for review**<br>- 165 duplicates (manually and automatically) identified and excluded<br>- 62 excluded as not related to DHIS2		**301**	
	**Full-text articles review**		**74**	

(b) PRISMA diagram

The literature search identified 301 research articles, of which 17 had a specific focus on DHIS2 in Kenya, and 67 wider research articles on DHIS2 from other Sub-Saharan countries or studies from Kenya with a minor focus on DHIS2 but reporting on neonatal data collection or ICT use. These included 42 research articles from other LMIC and 24 grey literature documents. Furthermore, 23 policy documents (see Supplementary file 2) from the Ministry of Health were reviewed for the narrative, in particular for the mapping.

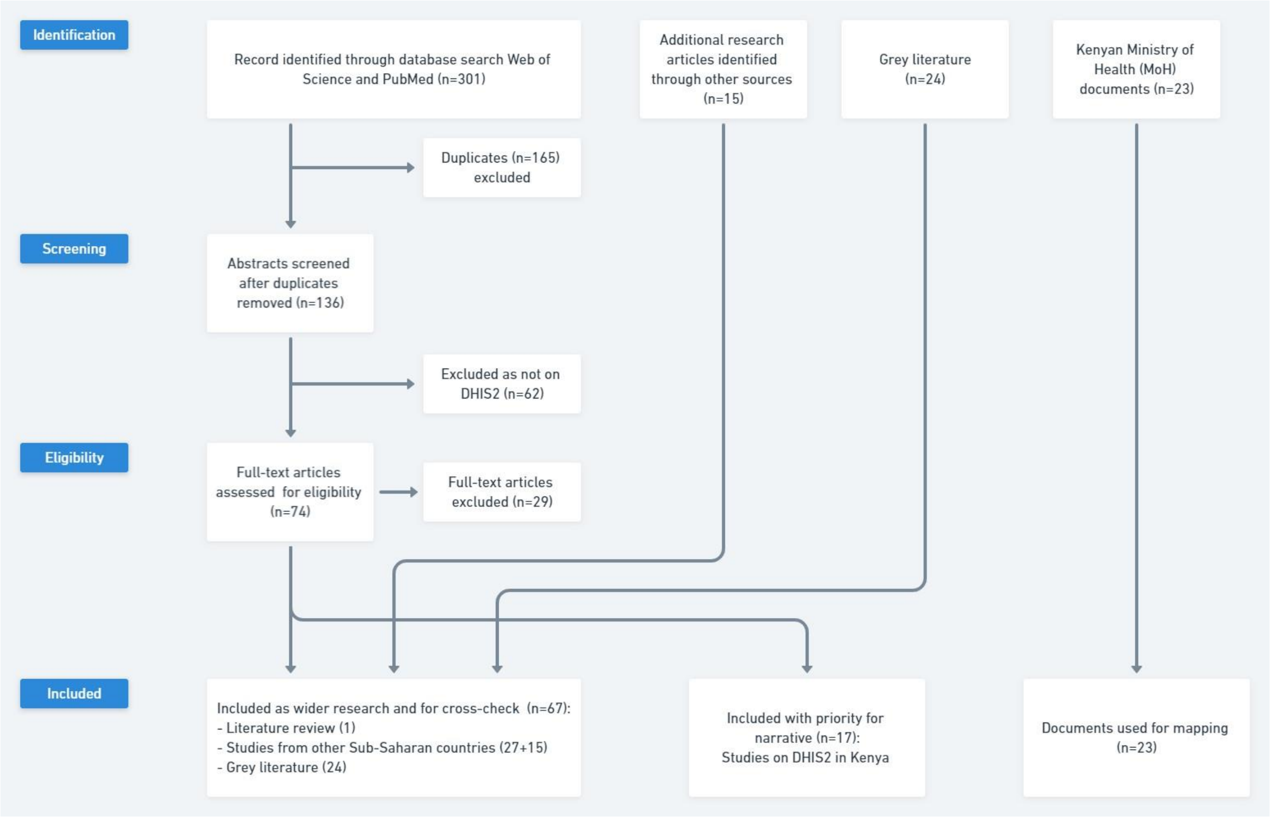

(c) Information flow diagram steps

Steps	Tasks
**1. Identification**	a) Search through text for details how information is supposed to flow<br>b) Search for other sources feeding into that system, receivers, and how information is supposed to flow (e.g. signal words like data reporting, collection, submission etc.)
**2. Collation**	a) Collate findings and sketch system around central information source as starting point (here: DHIS2)<br>b) If information does not fit into one generic flow, put identified information flows on top of each other and find generic flow
**3. Validation**	a) Share results with key informants working within the identified information system to validate results<br>b) Add, remove, change accordingly (if generic)
