# Supplementary material for: Data for tracking SDGs: challenges in capturing neonatal data from hospitals in Kenya
Source: BMJ Glob Health. 2020 Mar 31;5(3):e002108. doi: 10.1136/bmjgh-2019-002108 (PMC7170465; doi:10.1136/bmjgh-2019-002108)
Supplement: Supplementary data [file bmjgh-2019-002108supp002.pdf]

Supplementary file 2: Selected articles on DHIS2 in Kenya (a) and identified policy documents (b)

## (a) Selected articles on DHIS2 in Kenya

| # | AUTHOR (year) | TITLE |
|---|---|---|
| **1** | Manya et al. (2012) | National Roll out of District Health Information Software (DHIS2) in Kenya, 2011 – Central Server and Cloud based Infrastructure |
| **2** | Karuri et al. (2013) | Adoption of Health Information Systems by Health Workers in Developing Countries - Contextualizing UTAUT |
| **3** | Karuri et al. (2014) | DHIS2: The Tool to Improve Health Data Demand and Use in Kenya |
| **4** | Kihuba et al. (2014) | Assessing the ability of health information systems in hospitals to support evidence-informed decisions in Kenya |
| **5** | Manya & Nielsen (2015) | The Use of Social Learning Systems in Implementing a Web-Based Routine Health Information System in Kenya |
| **6** | Oluoch et al. (2015) | Do interoperable national information systems enhance availability of data to assess the effect of scale-up of HIV services on health workforce deployment in resource-limited countries? |
| **7** | Kariuki et al. (2016) | Automating indicator data reporting from health facility EMR to a national aggregate data system in Kenya: An Interoperability field-test using OpenMRS and DHIS2 |
| **8** | Tuti et al. (2016) | Improving documentation of clinical care within a clinical information network: an essential initial step in efforts to understand and improve care in Kenyan hospitals |
| **9** | Githinji et al. (2017) | Completeness of malaria indicator data reporting via the District Health Information Software 2 in Kenya, 2011 - 2015 |
| **10** | Maina et al. (2017) | Using health-facility data to assess subnational coverage of maternal and child health indicators, Kenya |
| **11** | Maina et al. (2017) | Coverage of routine reporting on malaria parasitological testing in Kenya, 2015 - 2016 |
| **12** | Okello et al. (2018) | "Every day they keep adding new tools but they don't take any away": Producing indicators for intermittent preventive treatment for malaria in pregnancy (IPTp) from routine data in Kenya |
| **13** | Kuyo at al. (2018) | Organizational Factors Influencing the Adoption of the District Health Information System 2 in Uasin GIshu County, Kenya |
| **14** | Manya et al. (2018) | Understanding the Effects of Decentralization on Health Information Systems in Developing Countries: A Case of Devolution in Kenya |
| **15** | Karuri et al. (2018) | Perceived Impact of Devolved Health Services on Implementation of DHIS2 in Kenya: A Qualitative Study |
| **16** | Muinga et al. (2018) | Implementing Open Source Electronic Health Record System in Kenyan Health Care Facilities: Case Study |
| **17** | Burnett at al. (2019) | Introduction and Evaluation of an Electronic Tool for Improved Data Quality and Data Use during Malaria Case Management Supportive Supervision |

## (b) Identified policy documents

Published national health policy and strategy documents from 2010 – 2018 were collated from the website of the Kenyan Ministry of Health - earlier also called Ministry of Medical Services/ Ministry of Public Health and Sanitation (MMS/MPHS) - and reviewed.

| # | PUBLICATION DATE | DOCUMENT TITLE |
|---|---|---|
| **23** | 2019 | Policy Brief - Enhancing Health Information System for Evidence based decision making in the Health Sector |
| **22** | 2013 | Report on the Baseline Assessment of Capacity for Monitoring and Evaluation |
| **21** | 2010 *presumably* | Standards and Guidelines for Electronic Medical Record Systems in Kenya |
| **20** | 2018 *presumably* | Health Sector Monitoring & Evaluation Framework 07/2014 – 06/2018 |
| **19** | 04/2017 | Kenya Standards and Guidelines for mHealth Systems |
| **18** | 02/2017 | Health Infrastructure Norms and Standards |
| **17** | 2016 | Kenya National eHealth Policy 2016-2030 |
| **16** | 05/2016 | One Monitoring and Evaluation Framework for the Health Sector in Kenya |
| **15** | 2016 | The Kenya Health Enterprise Architecture (KHEA) |
| **14** | 2015 | Kenya Health Workforce Report: The Status of Healthcare Professionals in Kenya, 2015 |
| **13** | 2014 | Kenya Health Policy 2014-2030 |
| **12** | 12/2014 | Health sector Human Resources Strategy 2014-2018 |
| **11** | 09/2014 | An Assessment Report for Hospitals |
| **10** | 2014 | Kenya Health Sector Data Quality Assurance Protocol |
| **9** | 2014 | Analysis of Performance, 2013/14 |
| **8** | 02/2013 | NHIS Infrastructure Assessment Report |
| **7** | 02/2013 | Implementing the District Health Information Systems v.2 |
| **6** | 2013 | Kenya Service Availability and Readiness Assessment Mapping (SARAM) Report |
| **5** | 2012 | The Kenya Health Sector Strategic and Investment Plan (KHSSP) |
| **4** | 05/2011 | Kenya Service Provision Assessment Survey 2010 (KSPA) |
| **3** | 04/2011 | Kenya National e-Health Strategy 2011 – 2017 |
| **2** | 2011 | Report on the Review of EMR Systems Towards Standardization |
| **1** | 2010 | Health Information System Policy 2010 -2030 |
